# Association study between a polymorphic poly-T repeat sequence in the promoter of the somatostatin gene and metabolic syndrome

**DOI:** 10.1186/s12881-018-0641-6

**Published:** 2018-07-27

**Authors:** Monique Tremblay, Diane Brisson, Daniel Gaudet

**Affiliations:** Lipidology Unit, Community Genomic Medicine Center, Department of Medicine, Université de Montréal and ECOGENE-21 Clinical and Translational Research Center, 225 St-Vallier Chicoutimi, Québec, G7H 7P2 Canada

**Keywords:** Somatostatin, Poly-T repeat, Gene polymorphism, Metabolic syndrome

## Abstract

**Background:**

Metabolic syndrome is a cluster of factors associated with an increased risk of developing type 2 diabetes mellitus (T2D) and coronary artery disease (CAD). It is a complex disorder resulting from the interaction between various environmental factors and genetic susceptibility. The somatostatin (SST) gene has been shown to regulate a wide range of functions, particularly in energy homeostasis. In addition, low levels of SST have been reported to have effects on the progression of metabolic syndrome components. The aim of this study was therefore to evaluate the association between polymorphic T sequences in the promoter of the SST gene and metabolic syndrome expression.

**Methods:**

We studied 1725 French-Canadian subjects from a founder population selected on the basis of having a positive family history of dyslipidemia, CAD or T2D. The analysis were performed on four groups created according to the poly T polymorphism length in the 5′ flanking promoter region of SST. Anova, Ancova and logistic regression models and Chi 2 analyses were used to evaluate the association between the poly T polymorphisms and metabolic syndrome components expression.

**Results:**

Analyses showed that means, frequencies and odds ratio of metabolic syndrome components expression increase as the number of poly-T repeats in the promoter region of SST increases. Women exhibit more significant differences than men. However, the trends are the same in both genders and differences for most of the components are significant in the entire sample.

**Conclusion:**

Those results suggest that the poly T polymorphisms in the SST promoter region may influence several metabolic processes implicated in metabolic syndrome expression. More analyses are needed to document the mechanisms that could underlie genetic regulation effect of SST on metabolic syndrome components and to clarify its specific role.

## Background

Metabolic syndrome is a cluster of interrelated common clinical disorders occurring together more often than by chance alone. It includes hyperglycaemia, hypertension, elevated triglyceride (TG) levels, low high-density lipoprotein (HDL) cholesterol levels and central obesity. The presence of 3 out of 5 risk factors indicates the presence of metabolic syndrome [1]. Metabolic syndrome has received worldwide attention for the past several years because of its increasing prevalence, which now ranges from 20% to nearly 35% of the adult population in almost all Western countries and, increasingly, in young age groups [2]. It has been estimated that people with metabolic syndrome are at twice the risk of developing coronary artery disease (CAD), compared with those without the syndrome, and experience a five-fold increased risk of type 2 diabetes (T2D) [1].

Numerous genome-wide association studies (GWAS) have been carried out for many common diseases, including metabolic syndrome and related covariables [3, 4]. More than 2000 robust association studies were published only between 2005 and 2012 [5]. Unfortunately, most of the gene variants identified usually explain only a small fraction of the heritability of these diseases, so that the value of such analyses is often met by scepticism. However, GWAS are not solely useful for identifying risk factors. Combined with functional studies and gene-environment interaction analyses, the mapping of gene networks associated with complex diseases, such as metabolic syndrome, may be essential to unveiling their aetiology and biological mechanisms [4].

Previous linkage analyses and association studies led us to target the somatostatin (SST) gene as a potential candidate with a significant position in the metabolic syndrome-associated gene network. We previously found a poly-T repeat sequence (rs34872250) in its promoter and documented an association between the length of the repeat and increased risk of hypertension, especially among women and overweight/obese individuals [6].

SST is a ubiquitous peptide that exerts effective inhibition of growth hormone (GH) release. It influences somatic growth and body weight by regulating the intestinal absorption of nutrients, gastro-intestinal motility, food intake and energy homeostasis. It also inhibits the production of insulin and glucagon, and limits the absorption of carbohydrates and TG in the small intestine. SST analogue has been used as therapy in the management of obesity and T2D. SST also inhibits a variety of other physiological functions in the central and peripheral nervous system, in the gastrointestinal tract and in the pancreas. Among its various activities, SST acts as a neurohormone and a neurotransmitter, and exercises autocrine and paracrine functions [7–9].

Considering the association between SST and numerous pathways well known to be involved in metabolic syndrome aetiology, as well as the central place of hypertension and overweight/obesity in its expression, it appears indispensable to investigate the association between poly-T repeat polymorphism and metabolic syndrome expression.

The aim of this study was therefore to investigate the relationship between the poly-T repeat polymorphism in the promoter of the SST gene and the expression of metabolic syndrome components.

## Methods

### Subjects and clinical data

This study comprised a sample of 1725 French-Canadian subjects from the Saguenay-Lac-Saint-Jean region of Quebec (Canada), selected on the basis of having a positive family history of dyslipidemia or CAD. All subjects had agreed to participate in studies on genetic determinants of T2D and/or CAD combining GWAS and candidate gene strategies [10, 11]. To be included in the analyses, all subjects had to be carriers of one of the four SST poly-T genotypes (13/13, 13/14, 13/15 and 13/16), as previously described [6].

The presence of 3 or more of the following components was used for the diagnosis of metabolic syndrome: waist circumference > 102 cm in men or > 88 cm in women (abdominal obesity); TG ≥ 1.7 mmol/L (hypertriglyceridemia); HDL-cholesterol < 1.03 mmol/L in men or < 1.3 mmol/L in women (low HDL-cholesterol levels); blood pressure ≥ 130 mmHg for systolic blood pressure or ≥ 85 mmHg for diastolic blood pressure or hypertensive treatment (hypertension); fasting glucose > 5.6 mmol/L, or drug treatment for elevated glucose (hyperglycaemia) [1]. T2D was defined according to the World Health Organization criteria as a 2-h glucose concentration > 11.1 mmol/L following a 75 g oral glucose load, whereas a normal glucose tolerance state was characterized as a 2-h glucose concentration below 7.8 mmol/L. Subjects with fasting TG > 20 mmol/L or BMI > 40 kg/m^2^, those taking drugs known to affect blood lipid levels and those known for abusive alcohol consumption were excluded. Body weight, height, and waist girth were determined according to the procedures of the Airlie conference [12]. Subjects gave their informed consent to participate in this study and were assigned a code that systematically de-identifies all clinical data [13]. This project received the approval of the Chicoutimi Hospital Ethics Committee and was conducted in accordance with the Declaration of Helsinki.

### Biochemical analysis

Blood samples were obtained after a 12-h overnight fast from the cephalic vein into vacutainer tubes containing EDTA. The HDL subfraction was obtained after precipitation of LDL (d > 1.006 g/ml) in the infranatant with heparin and MnCl_2_ [14]. Plasma glycerol concentrations were measured with a Technicon RA-500 analyzer (Bayer Corporation), and enzymatic reagents were obtained from Randox (Randox Laboratories). Glucose, free fatty acid and TG levels were measured with enzymatic assays on a CX7 analyzer (Beckman, Fullerton, CA, USA) [15]. Plasma LDL cholesterol levels were estimated using the Friedewald formula [16]. When TG levels were higher than 4.5 mmol/L, plasma LDL-cholesterol levels were calculated using a validated method [17].

### Microsatellite analysis

The length of the poly-T sequence was determined by DNA sequencing, using the BigDye™ terminator kit (Applied Biosystems Instruments, ABI, Foster City, CA, USA). The size standard was produced by PCR amplification of each individual clone identified with a fluorescent marker. Sample electrophoresis and data analysis PCR products were resolved and detected by capillary electrophoresis, using an ABI PRISM 3100 Genetic Analyzer, a multi-colour fluorescence-based DNA analysis system with 16 capillaries operating in parallel, with denaturing polymer POP4 (Perkin-Elmer, Shelton, CT, USA). Fragment sizing was supported using the Genescan™ (ABI, Foster Citym CA, USA).

### Statistical analysis

Continuous variables were compared using analyses of variance (ANOVA) followed by the Bonferroni post hoc test and analyses of covariance (ANCOVA) including age and gender as covariates, or the non-parametric Kruskal-Wallis and Mann–Whitney U tests. Variables with skewed distribution were log_10_-transformed before analyses and geometrical means are presented. Chi square (χ2) statistics were used to analyze distributions of categorical variables. Binary logistic regression models were constructed in order to calculate the relative odds of exhibiting components of metabolic syndrome according to the SST poly-T polymorphism genotype. The 13/13 genotype was considered as the reference group to which an odds ratio (OR) of one was set for comparison purposes. *P*-values were two-sided. All statistical analyses were performed with the SPSS package (releases 11.5 or 21, SPSS, Chicago Ill, USA).

## Results

The subjects’ characteristics are shown in Table 1. In the entire sample, significant differences (*p* <  0.01) were observed between the 13/13 and 13/16 genotypes for TG levels, waist girth, BMI and systolic blood pressure. There were also significant differences in systolic blood pressure levels between 13/14 and 13/16, and between 13/13 and 13/15 subjects. The tendency of TG levels to increase with the rising number of poly-T repeats is also interesting to note. Slightly different results were observed according to gender. In women, differences in BMI, systolic and diastolic blood pressure between genotypes are significant and they follow the same trend for waist girth. In men, only differences in TG and HDL-cholesterol concentrations are significant. Results remain the same after the inclusion of age and gender as a covariate.

**Table 1:** Subjects’ characteristics according to the SST poly T repeat polymorphism genotype in the entire sample and according to gender

All subjects Genotype	13/13			13/14			13/15			13/16			p	p*
n	913			69			571			172				
Gender M/W %	52.0/48.0			55.1/44.9			50.6/49.4			57.6/42.4			0.42	
Age (years)	49.7	±	11.8	47.4	±	11.7	50.1	±	12.2	50.3	±	12.1	0.33	
CT (mmol/l)	5.96	±	1.60	6.01	±	1.85	6.00	±	1.68	6.33	±	2.01a	0.07	0.06
HDL-C (mmol/l)	1.20	±	0.45	1.14	±	0.41	1.20	±	0.47	1.11	±	0.44	0.07	0.17
LDL(mmol/l)	3.66	±	1.47	3.70	±	1.33	3.59	±	1.25	3.74	±	1.62	0.63	0.71
TG (mmol/l) ^a^	1.80	±	2.22	1.89	±	2.26	1.90	±	2.12	2.19	±	3.11 a	**0.007**	**0.01**
WG (cm)	90.1	±	13.1	92.3	±	13.5	90.6	±	13.0	94.0	±	13.6 ac	**0.004**	**0.009**
BMI (kg/m^2^)	26.8	±	4.5	28.0	±	5.0	27.0	±	4.5	28.0	±	4.5 ac	**0.002**	**0.003**
Glycaemia (mmol/l) ^a^	5.58	±	1.49	5.87	±	1.75	5.67	±	1.68	5.82	±	1.56	0.06	0.05
Systolic BP (mmHg)	124.9	±	19.4	122.2	±	15.9	128.5	±	20.5	131.1	±	22.5 ab	**0.001**	**< 0.001**
Diastolic BP (mmHg)	78.4	±	11.5	79.0	±	10.9	79.2	±	11.1	80.9	±	11.4 a	0.05	0.08
FFA (mmol/l) ^a^	0.49	±	0.25	0.45	±	0.24	0.50	±	0.33	0.53	±	0.28	0.14	0.12
Glycerol (mmol/l) ^a^	0.07	±	0.09	0.06	±	0.03	0.07	±	0.28	0.07	±	0.06	0.52	0.28
Men														
Genotype	13/13			13/14			13/15			13/16			p	p**
n	475			38			289			99				
Age (years)	49.4	±	11.2	48.2	±	11.2	48.8	±	11.7	49.9	±	11.4	0.74	
CT (mmol/l)	5.93	±	1.57	6.17	±	1.43	5.98	±	1.61	6.35	±	2.14	0.12	0.106
HDL-C (mmol/l)	1.04	±	0.34	1.01	±	0.33	1.02	±	0.31	0.94	±	0.28 a	**0.036**	**0.034**
LDL(mmol/l)	3.73	±	1.46	4.03	±	1.50	3.73	±	1.30	3.79	±	1.71	0.67	0.68
TG (mmol/l) ^a^	1.90	±	2.30	2.08	±	2.29	2.00	±	2.12	2.44	±	3.61 a	**0.009**	**0.008**
WG (cm)	96.3	±	10.4	96.8	±	11.4	96.1	±	11.0	99.1	±	11.5	0.09	0.10
BMI (kg/m^2^)	27.4	±	3.9	28.1	±	4.2	27.4	±	4.0	28.2	±	4.1	0.23	0.22
Glycaemia (mmol/l) ^a^	5.77	±	1.64	6.31	±	2.12	5.74	±	1.48	5.87	±	1.31	0.11	0.09
Systolic BP (mmHg)	125.6	±	17.0	121.9	±	17.9	127.3	±	18.9	127.7	±	18.5	0.17	0.19
Diastolic BP (mmHg)	80.1	±	11.1	79.2	±	11.3	79.4	±	10.4	80.6	±	10.9	0.73	0.78
FFA (mmol/l) ^a^	0.43	±	0.24	0.40	±	0.24	0.46	±	0.29	0.49	±	0.27	0.07	0.17
Glycerol (mmol/l) ^a^	0.06	±	0.07	0.06	±	0.03	0.10	±	0.38	0.07	±	0.06	0.37	0.39
Women														
Genotype	13/13			13/14			13/15			13/16			p	p**
n	438			31			282			73				
Age (years)	50.1	±	12.4	46.5	±	12.3	51.4	±	12.7	50.9	±	13.0	0.16	
CT (mmol/l)	5.99	±	1.63	5.82	±	2.27	6.02	±	1.76	6.31	±	1.83	0.45	0.49
HDL-C (mmol/l)	1.37	±	0.48	1.31	±	0.44	1.38	±	0.53	1.34	±	0.51	0.83	0.88
LDL(mmol/l)	3.57	±	1.48	3.30	±	0.96	3.44	±	1.17	3.67	±	1.50	0.37	0.37
TG (mmol/l) ^a^	1.69	±	2.11	1.68	±	2.23	1.80	±	2.12	1.88	±	2.15	0.49	0.63
WG (cm)	83.5	±	12.6	86.7	±	13.9	85.0	±	12.4	87.0	±	13.2	0.07	0.05
BMI (kg/m^2^)	26.1	±	4.9	27.9	±	6.0	26.5	±	4.8	27.7	±	5.1	**0.019**	**0.013**
Glycaemia (mmol/l) ^a^	5.37	±	1.28	5.34	±	0.91	5.60	±	1.87	5.74	±	1.86	0.13	0.16
Systolic BP (mmHg)	124.1	±	21.7	122.6	±	13.4	129.7	±	22.0a	135.8	±	26.5 a	**< 0.001**	**< 0.001**
Diastolic BP (mmHg)	76.5	±	11.6	78.7	±	10.5	78.9	±	11.8	81.3	±	12.2 a	**0.003**	**0.003**
FFA (mmol/l) ^a^	0.57	±	0.26	0.53	±	0.23	0.55	±	0.36	0.60	±	0.29	0.42	0.66
Glycerol (mmol/l) ^a^	0.09	±	0.11	0.08	±	0.03	0.08	±	0.08	0.09	±	0.05	0.28	0.13

All data are mean ± Standard deviation

Significantly different (p <  0.05) as compared to (a) 13/13; (b) to 13/14; (c) to 13/15

Significant *p*-value are in bold

*CT* cholesterol total*, HDL-C* high density lipoprotein-cholesterol*, LDL-C* light density lipoprotein-cholesterol, *TG* triglycerides, *WG* Waist girth, *BP* blood pressure, *BMI* body mass index, *FFA* free fatty acid

*age and gender included as a covariates

**age included as a covariate

^a^= geometrical mean and p-value obtained after log_10_ transformation

As shown in Table 2, the prevalence of abdominal obesity, hypertriglyceridemia, hypertension and metabolic syndrome are significantly different (*p* <  0.05) across SST poly-T repeat groups in the entire sample, whereas there was a tendency toward low HDL-cholesterol levels and hyperglycemia. In women, significant differences in the prevalence of hypertension and metabolic syndrome diagnosis were noted, and there was a tendency to develop abdominal obesity. In men, only hypertriglyceridemia prevalence was significantly different across the groups.

**Table 2:** Prevalence (%) of metabolic syndrome components according to the SST poly-T repeat polymorphism genotype

	Genotypes				
All subjects	13/13	13/14	13/15	13/16	p
n	913	69	571	172	
Abdominal Obesity	58.3	65.2	59.5	69.8	**0.032**
Hypertriglyceridemia	50.4	55.1	55.7	61.6	**0.026**
Low HDL-C level	51.2	50.7	51.5	62.2	0.058
Hypertension	33.6	23.2	39.2	42.4	**0.005**
Hyperglycaemia	36.5	46.4	38.2	45.9	0.060
Diagnosis of Metabolic Syndrome	46.8	47.8	49.6	61.6	**0.005**
Men					
n	475	38	289	99	
Abdominal Obesity	74.5	73.7	72.3	80.8	NS
Hypertriglyceridemia	53.3	63.2	60.2	68.7	**0.019**
Low HDL-C level	55.6	50.0	56.4	67.7	NS
Hypertension	33.3	21.1	34.6	35.4	NS
Hyperglycaemia	43.6	55.3	42.9	49.5	NS
Diagnosis of Metabolic Syndrome	57.1	52.6	56.7	66.7	NS
Women					
n	438	31	282	73	
Abdominal Obesity	40.6	54.8	46.5	54.8	0.056
Hypertriglyceridemia	47.3	45.2	51.1	52.1	NS
Low HDL-C level	46.3	51.6	46.5	54.8	NS
Hypertension	34.0	25.8	44.0	52.1	**0.002**
Hyperglycaemia	28.8	35.5	33.3	41.1	NS
Diagnosis of Metabolic Syndrome	35.6	41.9	42.2	54.8	**0.013**

NS: *p* ≥ 0.1 Abdominal obesity = waist circumference > 102 cm in men or > 88 cm in women; Hypertriglyceridemia = TG > 1.7 mmol/L; Low HDL-C level = HDL-Cholesterol < 1.03 mmol/L in men or < 1.3 mmol/L in women; Hypertension = blood pressure ≥ 130 mmHg systolic blood pressure or ≥ 85 mmHg diastolic blood pressure or hypertensive treatment; Hyperglycaemia = fasting glucose > 5.6 mmol/L or drug treatment for elevated blood glucose

Significant *p*-value are in bold

Table 3 shows various multivariate models, including age and gender as covariates, in which each components as well as the metabolic syndrome are one after the other the dependent variable. Obtained results confirmed the increased odds of exhibiting metabolic syndrome components associated with the 13/16 poly-T repeat genotype when compared with the 13/13 poly-T repeat genotype. In the entire sample, the risk was significantly increased for all metabolic syndrome components among subjects with the 13/16 genotype. In addition, the risks of hypertriglyceridemia and hypertension also increased significantly among the 13/15 carriers. Differences appear between genders, with the risk of hypertriglyceridemia and low HDL-cholesterol level being significant in men, while the risk of abdominal obesity, hypertension, hyperglycemia and metabolic syndrome are significant in women.

**Table 3:** Estimated relative risk (odds ratio) of exhibiting metabolic syndrome components associated with the SST poly-T repeat polymorphism genotype

	Genotypes		Abdominal Obesity	Hypertriglyceridemia	Low HDL-Cholesterol	Hypertension	Hyperglycaemia	Metabolic Syndrome
Entire sample^a^	13/13	p= OR	1	1	1	1	1	1
	13/14	OR	1.46	1.24	0.96	0.66	1.68	1.10
		*p*-value *95%CI*	0.18 (0.84–2.53)	0.39 (0.76–2.04)	0.87 (0.59–1.57)	0.18 (0.36–1.21)	*0.05* (1.01–2.81)	0.71 (0.66–1.83)
	13/15	OR	1.07	1.24	1.02	1.26	1.07	1.13
		p-value *95%CI*	0.57 (0.85–1.34)	*0.05* (1.01–1.53)	0.85 (0.83–1.26)	*0.05* (1.00–1.59)	0.53 (0.86–1.34)	0.28 (0.91–1.40)
	13/16	OR	1.60	1.55	1.55	1.48	1.43	1.79
		p-value *95%CI*	*0.01* (1.10–2.33)	*0.01* (1.11–2.17)	*0.01* (1.11–2.17)	*0.03* (1.04–2.11)	*0.04* (1.02–2.02)	*< 0.01* (1.27–2.53)
Men^b^	13/13	OR	1	1	1	1	1	1
	13/14	OR	0.99	1.49	0.79	0.55	1.71	0.85
		p-value *95%CI*	0.98 (0.46–2.12)	0.25 (0.75–2.96)	0.50 (0.41–1.54)	0.15 (0.24–1.25)	0.12 (0.87–3.37)	0.64 (0.66–1.83)
	13/15	OR	0.91	1.32	1.03	1.09	0.99	1.00
		p-value *95%CI*	0.58 (0.65–1.27)	0.07 (0.98–1.78)	0.84 (0.77–1.39)	0.59 (0.79–1.50)	0.96 (0.73–1.34)	1.00 (0.91–1.40)
	13/16	OR	1.43	1.93	1.68	1.08	1.26	1.50
		p-value *95%CI*	0.20 (0.83–2.47)	*0.01* (1.22–3.07)	*0.03* (1.06–2.65)	0.75 (0.68–1.72)	0.31 (0.81–1.96)	0.08 (0.95–2.37)
Women^b^	13/13	OR	1	1	1	1	1	1
	13/14	OR	2.08	1.04	1.21	0.86	1.66	1.59
		p-value *95%CI*	0.06 (0.98–4.42)	0.91 (0.49–2.21)	0.61 (0.58–2.52)	0.74 (0.35–2.11)	0.21 (0.75–3.67)	0.24 (0.74–3.43)
	13/15	OR	1.22	1.12	1.01	1.45	1.17	1.26
		p-value *95%CI*	0.21 (0.89–1.66)	0.49 (0.82–1.52)	0.94 (0.75–1.37)	*0.03* (1.03–2.04)	0.35 (0.84–1.64)	0.16 (0.91–1.73)
	13/16	OR	1.77	1.19	1.41	2.33	1.73	2.25
		p-value *95%CI*	*0.03* (1.06–2.95)	0.51 (0.72–1.98)	0.18 (0.86–2.32)	*< 0.01* (1.33–4.09)	*0.04* (1.02–2.93)	*< 0.01* (1.33–3.79)

^a^Including age and sex as covariates

^b^Including age as a covariate

Significant *p*-values were italicized

## Discussion

Our study shows that the length of the SST poly-T repeat polymorphism is significantly associated with the expression of metabolic syndrome components, as well as the metabolic syndrome diagnosis itself. Although effects of SST analogue administration on metabolic syndrome component expression have been previously studied, [9] to the best of our knowledge, this is the first study on the association between the SST genotype and metabolic syndrome.

The SST gene is located on chromosome 3q27, a quantitative trait loci (QTL) associated with multiple representative traits of metabolic syndrome. Several familial and population studies have confirmed the existence of links between this QTL and metabolic syndrome. A number of candidate genes associated with the expression of the latter have been found. However, those genetic factors can explain only a small fraction of the population variance. The full spectrum of contributing loci could be larger. The small part of each gene in the expression of this complex trait could result from the interaction between various genes, in addition to an interaction between genes and environmental factors [18].

SST is known to be associated with a variety of physiologic functions, among which many are involved in the expression of metabolic syndrome, including glucose metabolism [19]. However, according to our results, the differential expression of metabolic syndrome according to poly-T repeat length appears mainly due to its association with high blood pressure, dyslipidemia and obesity, while glycaemia homeostasis does not appear to be significantly affected.

As previously published, the SST poly-T repeat polymorphism is significantly associated with increased blood pressure and risk of hypertension, particularly in women and in overweight/obese individuals. Various pathways could explain this association. Among them, inhibition of the vasodilator glucagon, a decrease in plasma renin activity, facilitation of vasoconstrictors, or the relationship with neuronostatin may be involved in the rise of blood pressure [6].

Effects of SST on lipid levels have been only partially investigated and published conclusions contain apparent discrepancies. SST analogues administered in the context of acromegaly seem to inhibit GH release, with favourable changes in plasma lipids, and improvement of insulin sensitivity [20]. However, increased fat mass, an increase in TG level and reduced HDL-cholesterol levels have been observed in untreated GH deficiency. Moreover, some observations have shown that GH level is negatively associated with adipose tissue mass, while GH replacement has been shown to improve those factors in dose-dependant levels [21]. GH deficiency and acromegaly are at the opposite ends of the GH level spectrum. In acromegaly, the improvement of lipid profile with SST analogues might be the result of a reduction in GH secretion. However, in a non-acromegalic individual, increased SST output may suppress GH secretion and, as a result, may be the cause of GH deficiency. Thus, it can be hypothesized that increased SST secretion may cause hypertriglyceridemia and a low level of HDL-cholesterol [21].

Because the prevalence of abdominal obesity appears to rise with an increase of the poly-T repeat length, we cannot exclude the possibility that the obesity could, at least partially, cause the observed hypertriglyceridemia and low HDL-cholesterol levels. Although there are several reports showing that the administration of SST plays a role in eating behaviours and body weight, the results have been controversial, probably because of the multiple actions of this peptide on central and gastrointestinal functions [22]. Accordingly, sizeable ingestion of macronutrients, including carbohydrates, fat and protein can induce low-grade inflammation and reduced secretion of intestinal SST. These effects may in turn increase intestinal absorption of energy and nutrients and the formation of adipose tissues. The SST analogue octreotide has been shown to reverse this process in order to regulate the energy balance. SST may also act on obesity by limiting insulin release, which is the primary hormonal mediator of adipogenesis in humans [8]. Conversely, it has been reported that SST may modulate leptin signalling in hypothalamus and ghrelin circulation and then serves as an orexigenic neuropeptide [23]. Injections of SST agonist over long periods have reduced body weight gain and lean mass independently of food intake in lean rats. These effects are related to GH inhibition. However, the same SST agonist has stimulated increased food intake and fat mass in diet-induced obese rats. Indeed, several studies have indicated that obesity is associated with enhanced SST quantity and responsiveness and that inhibition of hypothalamic SST release consistently improves GH response in obese subjects [24]. Moreover, polymorphisms in the SST gene receptor have been associated with anthropometric variables and increased food intake. The relationship between SST and obesity does not appear to be simple, and recent evidence points to a divergent action of SST in the brain and in the periphery (gut, stomach, pancreas) [24].

These observations agree with our results. Indeed, mean concentrations of almost all the components studied increase from genotype 13/13 to 13/16. Although they are not all significant, these associations may contribute to the increase in metabolic syndrome elements, according to genotype. The features of metabolic syndrome are interrelated and share common pathways, and it is still not fully understood how these components interact over time, and what the real impact of genetics and the environment is on each of them. Thus, the effects of SST may be far more complex than initially expected, but their study may help to build a better understanding of the interplay among metabolic syndrome components.

We observed differences between men and women. SST regulates GH release and synthesis in a pulsatile pattern that is gender dependent. GH deficiency is associated with increased fat mass and lower lean body mass, metabolic derangements, including insulin resistance, and suboptimal physical performance [25]. Under a normal fed condition, GH release in males tends to be highly organized, but it is disorganized in females [26]. In the obese state, SST levels are elevated in the portal circulation, and systemic immunoneutralization of SST restores larger GH pulses. Thus, our current observations are in accordance with other studies, which demonstrate that SST output is more critical in females than males, but may be relevant for both of them in the case of abdominal obesity [27]. However, detailed studies examining the gender-dependent role SST plays in regulating GH pulse release in humans has not been directly studied and therefore caution should be exercised in extrapolating the results of experiments across species. Another possible explanation for these differences could be related to fat distribution. Usually, men present with more abdominal fat than women and thus, for a given excess of BMI, women may be less prone to developing other metabolic alterations [28]. This may explain that, while the means of WG and BMI are significant in women, the other components are not. However, we cannot exclude the possibility that those differences may also be influenced by other hormonal or environmental factors.

In this study, only slight phenotypic changes were observed, as in strains of knockout mouse created in order to study SST function [29]. Those results suggest that the poly-T repeat polymorphisms in the SST promoter region may influence several processes involved in metabolic syndrome, probably by acting as a fine-tuning regulator of energy metabolism. Even if the contribution to the variation differs between genders, it seems to follow the same trend. The hypothesis that an increase in the number of T repeats could act by increasing SST output is appealing, but more research is needed to better understand the processes that determine the genetic regulation of SST on metabolic syndrome components and particularly, to clarify its specific role.

The French-Canadian population of Saguenay-Lac-St-Jean, from which our subjects originate, is a strength in our study. This population descended from a founder population that settled in this region 300–400 years ago. This founder effect provides several benefits for mapping the genomic determinants of complex traits [30]. Genetic heterogeneity remains a problem in disease identification strategies that can be avoided by analyzing homogeneous populations with geographic stability, and which are most likely uniform in their environmental exposure [31].

Our study has however some limitations. All groups have an average BMI and waist circumference over the limit of overweight, except for waist girth among women with 13/13 genotypes. This could act as confounder because, as previously seen, abdominal obesity is well known to disrupt SST quantity and responsiveness. Another limitation is selection bias, because participants were recruited among lipid clinic patients. This may have led to a higher prevalence of alteration in lipid profile or components such as T2D. Moreover, we cannot exclude the possibility that other genetic polymorphisms may influence lipid profile and the results. Thus, it is essential to reproduce these analyses in other populations with different phenotypes.

## Conclusions

Our study suggests that the polymorphism in the SST gene promoter is associated with the onset of metabolic syndrome. Our results vary along gender lines but the same tendency seems to be found in both sexes. However, as noted, this study was performed among subjects issued from studies on genetic determinants of T2D and/or CAD, so that the proportion of subjects with normal metabolic profile is quite under what is observed in the general population. Obtained results therefore need to be confirmed among other samples, more representative of the general population.

## Abbreviations

ANCOVA: Analyse of covariance
ANOVA: Analyse of Variance
CAD: Coronary artery diseases
GH: Growth Hormone
GWAS: Genome-wide Association Studies
HDL: High density lipoprotein
SST: Somatostatin
T2D: Type 2 diabetes mellitus
TG: Triglycerides
χ2: Chi square
